# Same, but Different? Digital Transformation in Swiss Vocational Schools from the Perspectives of School Management and Teachers

**DOI:** 10.1007/s10758-022-09631-9

**Published:** 2022-12-05

**Authors:** Martina Rauseo, Andreas Harder, Deborah Glassey-Previdoli, Alberto Cattaneo, Stephan Schumann, Serge Imboden

**Affiliations:** 1 Swiss Federal University for Vocational Education and Training, Lugano, Switzerland; 2 University of Konstanz, Konstanz, Germany; 3 University of Applied Sciences HES-SO Valais-Wallis, Sierre, Switzerland

**Keywords:** Digital Transformation, Vocational Education, Switzerland, School Improvement, School Management, Teachers

## Abstract

The COVID-19 pandemic has further highlighted the importance of the digital transformation of educational organizations. The effects of this transformation process are not limited to the classroom level but extend into various areas of the school, such as *Equipment and Technology*, *Strategy and Leadership*, *Organization*, *Employees*, and *Culture*. Against this background, we assessed the status quo of the digital transformation in Swiss vocational schools from the perspective of both school management members and teachers. For this endeavor, two surveys were conducted: the first one in the period from November 2019 to January 2020 (school management members) and the second one between June and September 2020 (teachers). In total, 202 school management members and 1,739 teachers from 62 schools participated in the study. The results of the analyses indicate that the digitization-related dimensions of *Strategy and Leadership*, as well as *Pedagogical IT Support*, were perceived better by school management members, whereas teachers considered the areas of *Digital Competencies*, *Attitudes*, and *Culture* to be more advanced. Furthermore, linear regression models show that the dimensions considered important when assessing the status quo of digital transformation differ between the groups. The results emphasize the importance of communication between and the inclusion of all school stakeholders for the successful management of the digital transformation.

## Introduction

Digital transformation is a global trend that permeates and influences private life as well as the world of work (e.g., World Economic Forum, 2016; Arnold et al., 2016; Cijan et al., 2019). The term “digital transformation” describes the profound structural change of the working world through digital technologies such as mobile, networked devices, cloud computing, digital networking, social media, internet of things, big data, artificial intelligence, and robotics (Bosch et al., 2018). It leads to new business models, work processes, and job profiles, and therefore to new requirements regarding the competence profiles of employees and apprentices (Genner, 2017). In line with the broad transformation processes in the world of work, the digital transformation also encompasses extensive changes in the school sector. These changes are thematically diverse and complex and are not limited to technological aspects, such as the digital infrastructure or equipment of a school or a classroom, but also include, among other things, a digital school organization, curricular adaptations, other competence requirements for school management members and teachers, and generally an adaptation of the school culture. Only by taking this holistic view, the digital transformation can reach the teaching level in a sustainable way (Petko et al., 2018a). In this context, the Swiss Federal Council (2018) has developed a strategy for a digital Switzerland, with one of the pillars being the area of education.

Consequently, the Swiss State Secretariat for Education, Research and Innovation (SERI) (2017) compiled an action plan with the intention of enhancing competencies in education and research in Switzerland. The concept distinguishes between eight different dimensions, of which the first four areas are relevant for upper secondary education (including vocational schools): (1) improving digital literacy, (2) using information and communication technology (ICT) in teaching and learning, (3) rapidly adapting the education system to market requirements, and (4) improving coordination and communication in education cooperation. The primary objective of education in this field, particularly in vocational schools, is to enable students to acquire the digital competencies necessary to carry out their professional activities. Genner (2017) argued that, in the context of digital transformation, the dual training system (i.e., vocational training alternating a company-based and a school-based track) plays an important role in providing the necessary competencies due to its close connection between the educational institution, on the one hand and the working reality on the other hand.

A key factor in achieving these digitalization-related educational goals is teachers that have appropriate digital competencies (e.g., Petko et al., 2018b). Thus, the European Commission has launched various initiatives to assess the status quo regarding the digital competencies of teachers. Based on their theoretical competence framework – the Digital Competence Framework for Educators (DigCompEdu) – they developed a self-assessment tool, which again decomposes the construct of “digital competence” into six dimensions (Carretero et al., 2017; Redecker & Punie, 2017), further illustrating the scope and complexity of this concept.

However, digital transformation is not limited to the classroom level. The successful achievement of digitalization-related educational goals requires appropriate framework conditions in schools, which are considered complex institutions (e.g., Eickelmann et al., 2019). Therefore, it is important to take the impact of digital transformation on schools at various levels into account (Ifenthaler & Egloffstein, 2020). Petko et al. (2018b) addressed this topic by defining the concepts of *teacher readiness* and *school readiness* in the context of teachers’ use of ICT. Thus, in addition to the attitudes and competencies of the teachers *(teacher readiness)*, certain school-level conditions are also necessary; for example, sufficient technological infrastructure and equipment, functioning IT support, or the prioritization of the digital transformation in general *(school readiness)*. The International Computer and Information Literacy studies (ICILS), which focus on measuring the digital competencies of eighth graders, also highlight the need for appropriate school conditions and emphasize the special role of school management in this context (Fraillon et al., 2014, 2020). School management, in fact, is primarily responsible for initiating and implementing school development processes and can help ensure that the framework conditions are conducive through its leadership behavior (e.g., Bonsen 2016a).

In addition to an increasing trend toward digitalization, the COVID-19 pandemic has accelerated this development even further. Following the Federal Council’s (2020) decision of school closures in the spring of 2020, the schools only had a few days to propose an appropriate solution for the following weeks and months, which has led to challenges for both school management and teachers. From a scientific point of view, the school closures represent a situation in which the digitalization of schools can be observed in a very special context. In some studies, this situation was the basis for examining how well schools were digitally positioned to deal with the pandemic-related challenges at that point in time. The pandemic can therefore be seen as a trigger that has once again brought digital transformation into sharper focus.

In summary, both school management and teachers play important roles in achieving digitalization-related educational goals and supporting the digital transformation of the educational system. Whereas school management should ensure the necessary framework conditions and initiate and design corresponding school development processes, teachers are responsible in the classroom for ensuring that learners are prepared for the digitalized world of work in the best possible way. In this context, ICT’s potential for teachers in the classroom is multifaceted: on the one hand, ICT can foster pedagogical diversity of methods for teaching subject-specific content, and on the other hand, the development of digital competencies is promoted through critical engagement with them. However, to the best of our knowledge, there are no studies investigating how these two points of view – the school management members’ and the teachers’ – position themselves in the process of digital transformation, especially regarding vocational education. Therefore, this study focuses on the perceptions of school management members as well as teachers regarding various aspects of digital transformation to identify the potential for optimization and to derive evidence-based, appropriate recommendations for practice.

## Theoretical Background

The first part of this section presents the advantages and disadvantages of different static model approaches in the context of digital transformation. Static models allow us to assess the status quo of educational institutions at a specific point in time. However, since digital transformation is in doubt about long-term development processes, a dynamic view is also essential to classify the development status of the respective schools. This circumstance will be briefly addressed in the second part of the section.

Development processes in schools are not limited to the classroom level. Rolff’s (2010) triad of school improvement is one of the most prominent models dealing with this topic, at least in German-speaking countries. In this context, organizational, personnel and instructional development are defined as the three core dimensions of school improvement. In the light of digitalization, several models have been developed to approach the topic from a school improvement perspective and to define relevant content areas for this field (Waffner, 2021). Rolff’s (2010) general approach was then further expanded by including the dimensions concerning technology and cooperation development (Schulz-Zander, 1999; Eickelmann & Gerick, 2017).

Another well-known model focusing on the implementation of good practices within a school and its relevant stakeholders is the so-called Innovative Digital School (IDI) model (Ilomäki & Lakkala, 2018), in which the pedagogical and school-level knowledge practices as well as the digital resources form the foundation of the digital development of the school. Furthermore, the practices of the teaching community on the one hand, as well as the vision of the school in connection with adequate leadership on the other hand, are necessary for successfully shaping digital development processes. Although this approach focuses predominantly on the practices of the different stakeholders, organizational, school cultural, or strategic elements of digital transformation are addressed only implicitly.

A model that explicitly addresses the relatively abstract approach of Rolff (2010) as well as the rather low differentiation of organizational and personal resources of the IDI model (Ilomäki & Lakkala, 2018) is the so-called Maturity Model for Educational Organizations (MMOE) (Ifenthaler & Egloffstein, 2020). The MMOE does not put the specific practices in the schools in the foreground but tries to define areas to determine the digital status quo of an organization. Although this model originally does not explicitly refer to schools but to educational organizations in general, it can be used as a holistic concretization of the abstract dimensions of the previously mentioned models, with a special focus on digital transformation. The MMOE outlines six main dimensions: *Equipment and Technology*, *Strategy and Leadership*, *Organization*, *Employees*, *Culture*, and *Digital learning and teaching*. Based on these parameters, it is possible to determine how ‘mature’ the school organization is in terms of digital transformation. In the second step, these dimensions are then operationalized with several indicators that illustrate the broad spectrum of some of the dimensions. A visualization of the model and its components is presented in Table 1.

**Table 1:** Dimensions of the Maturity Model for Educational Organizations (Ifenthaler & Egloffstein, 2020)

Dimension	Indicators/content
*Equipment and Technology*	Equipment with digital devices, software Up-to-date infrastructure Homogeneous technology landscape, standards
*Strategy and Leadership*	Existence and implementation of a digital strategy Managers promote digitalization with priority Analysis of new technologies Democratic leadership style, creative freedom granted
*Organization*	Sufficient financial resources Technical support (internal vs. external service providers) Efficient procurement and maintenance Pedagogical support
*Employees*	Knowledge/Skills in dealing with digital technologies Usage of devices and services Attitudes Readiness for further training
*Culture*	Openness to new technologies Openness for change Open communication, mutual support
*Digital learning and teaching*	Digital platforms, e-learning offerings Working with digital devices in classroom settings Digital education as an overall goal Data-driven teaching and learning

Regardless of the question of which of these models depicts digital school improvement best, all approaches mentioned above can be classified as static, as they primarily aim to delineate relevant dimensions and do not focus process-related aspects in the first place. In addition to these static models, there are some approaches that attempt to map school improvement processes against the backdrop of digital transformation from a dynamic perspective. For example, Gräsel et al. (2020) defined input, processual, and output factors based on existing school improvement models to successfully shape corresponding digital development processes. Classic change management approaches from the world of work can also help in this context, serving as a theoretical basis for the successful implementation of these models (e.g., Kotter 2012).

In both static and dynamic models, all school stakeholders – that is, school management members, teachers, and students – contribute to the successful implementation of these development processes (e.g., Ilomäki & Lakkala, 2018). At the individual school level, teachers are directly responsible for building digital literacy among students, whereas school management takes on a special role by initiating and sustainably shaping the framework conditions and setting the right direction. This was corroborated by Petko et al. (2018b), who stated that technology integration depends on *teacher readiness*, which in turn is influenced by *school readiness*. From a more practical perspective, a specialized agency commissioned by the Swiss Confederation and the cantons, called Educa (2021), published a report that also addresses the important factors that enable the successful implementation of digital resources. Among other things, the report states that a strategy implemented by the school in the long term has a positive effect on school success. Furthermore, the report emphasizes the centrality of teachers’ beliefs and competence in the use of ICT in learning and teaching. This must also be fostered by a school environment that promotes digitalization (Educa, 2021). These elements can again be found in the MMOE (Ifenthaler & Egloffstein, 2020).

Regarding the COVID-19 crisis, empirical results indicate that, in entering the crisis, schools had different starting positions in terms of digital prerequisites; in other words, the *readiness* of some schools was better developed than that of other schools (Feldhoff et al., 2021). The respective schools were able to deal better with the various challenges caused by the pandemic. If we compare the situation in the German-speaking countries of Switzerland, Germany, and Austria, studies show that Swiss schools were comparatively better prepared in terms of digital resources than schools in the other two countries (Huber et al., 2020), although the general level of teachers’ digital competence aligned with the international average (Cattaneo et al., 2022). These findings are also confirmed by the longitudinal S-Clever study, which surveyed school management members in all three countries, although the school management members addressed the challenges of the pandemic in similar ways. However, the perception of specific challenges shows that the field of digital transformation is still topical and has not yet been settled by the pandemic, since there have been no significant improvements over time in dealing with teachers’ emotional or motivational burdens or the digital equipment in schools in all three countries (Feldhoff et al., 2022).

In summary, the different approaches and ideas demonstrate the complexity of school improvement, especially regarding digital transformation. Furthermore, the indispensable necessity to further develop schools digitally becomes apparent. In this context, the COVID-19 crisis has served as an eye-opener for many schools.

## Research Question

In the context of the SERI action plan (2017), the first focus of our study lies on the general state of development of digital transformation of vocational schools in Switzerland. In the next step, we refer to the portrayed MMOE, which shows from a theoretical perspective how multilayered and differentiated the digital transformation in the school context is. However, whether and to what extent the various dimensions actually contribute to the perception of the digitization-related status quo remains open. In line with these theoretical considerations, three central research questions can be derived:

1. What is the status quo of the general state of development of digital transformation in Swiss vocational schools from the perspective of both school management members and teachers?
2. Are there differences in the perception of the various sub-areas of digital transformation in schools between the two groups?
3. Which of these sub-areas are predictive of the perceived digitalization-related state of development?

Many studies examine the digital transformation from the perspective of one particular school actor, i.e., either the school management, the teachers, or the learners. The content-related focus of these studies often differs: While school management members are more likely to be asked questions about organizational development, studies with teachers often deal with elements of instructional or classroom development. Since the successful design of school development processes in general – and the digital transformation in particular – requires the sustained participation of all school stakeholders, we will examine whether differences can be identified between school management members and teachers in terms of their perceptions regarding all three research questions. The principal aim of our study is to combine the two perspectives and thus to obtain a broader picture of the digitalization-related status quo from both groups. On this basis, implications that can support vocational schools in successfully shaping school development processes regarding digital transformation will be developed.

## Methods

### Design

The presented study is the result of a cooperation between the Swiss Federal University for Vocational Education and Training (SFUVET), the University of Applied Sciences HES-SO Valais-Wallis and the University of Konstanz from Germany. It is based on a collaboration between two existing projects conducted independently by the University of Applied Sciences HES-SO Valais-Wallis and the University of Konstanz on the one hand, and SFUVET on the other.

Both projects are based on cross-sectional surveys which assess the various dimensions of digital transformation from the perspectives of the two groups (school management and teachers). The survey of the first project was conducted from November 2019 to January 2020, and therefore immediately before the start of the COVID-19 pandemic in Europe. Among other things, the selection of these schools relied on the *Smart School Competition 2019* of the German digital association *Bitkom e.V.* (Harder et al., 2020). Given that the composition of the school management team can vary within schools, the teams were allowed to decide who would be considered a school management member in their schools and therefore participate in the survey.

The second project by SFUVET was launched in January 2020. The main goal was to assess the digital competencies of teachers in vocational education and training in Switzerland. The survey took place from June to September 2020 and was primarily based on the European DigCompEdu Framework (Carretero et al., 2017; Redecker & Punie, 2017).

Since the cooperation between all three institutions started in April 2020, we were able to integrate several items of the instrument used for the school management members in the survey for the teachers. However, due to the different orientations of the projects and organizational reasons (i.e., the length of the instrument), the number of possible common items was limited.

### Sample

According to the Federal Statistical Office (2021), there are 382 public schools for vocational education and training in Switzerland which employed 15,698 teachers (57.6% male) in the school year 2019/2020. There are no specific figures for the vocational education sector in terms of the number of extended school management members.

A total of 202 school management members and 1,739 teachers from 62 vocational schools from the three main language regions in Switzerland voluntarily and anonymously participated in both cross-sectional surveys. Regarding the population, this corresponds to a rate of 16.2% of Swiss vocational schools and 11.1% of the teachers employed there. Furthermore, the majority of the participants lived in the German-speaking part of Switzerland (68%), followed by the French (20%) and Italian (12%) language regions. We also found a significantly larger proportion of male participants among school management members (80%), while gender was roughly balanced among teachers. A detailed sample description is presented in Table 2.

**Table 2:** Sample description

		School Management (SM) n = 202		Teachers (T) n = 1.739		Total	
		*Male*	*Female*	*Male*	*Female*		
**Language Region**	*German* ^*1*^	100 (50%)	31 (15%)	539 (33%)	556 (35%)		1.226 (68%)
	*French* ^*2*^	42 (21%)	9 (4%)	176 (11%)	142 (9%)		369 (20%)
	*Italian* ^*3*^	18 (9%)	1 (1%)	75 (5%)	121 (7%)		215 (12%)
**Total**		160 (80%)	41 (20%)	790 (49%)	819 (51%)		1.810 (100%)

*Note.* Deviations result from missing values (n = 1.942)

^1^ Argovia, Basel-Country, Basel-City, Berne, Glarus, Grisons, Lucerne, Nidwald, Obwald, Schwyz, Soleure, St. Gall, Thurgovia, Uri, Zoug, Zurich

^2^ Friburg, Geneva, Jura, Neuchatel, Vaud, Wallis

^3^ Tessin

### Instrument

In line with the research questions, our goal was to assess the status quo of digital transformation from the perspectives of school management members and teachers. In view of the items that the two surveys had in common, the MMOE (Ifenthaler & Egloffstein, 2020) was chosen as the framework for the study, which became the common denominator on which the analyses were conducted.

The instrument used in the school management survey, which had already attempted to cover the broad spectrum of digital transformation as well as possible, served as the starting point for the design of the joint instrument. Following the teacher survey and the subsequent data processing, an attempt was made to summarize the items in a plausible way in terms of content using an exploratory factor analysis. This resulted in some dimensions being represented by only one item, while others were operationalized with multiple scales. In the second step, the internal consistency of the potential scales was tested. Afterwards, individual items, as well as the newly formed scales, could be assigned to the superordinate dimensions of the MMOE (Ifenthaler & Egloffstein, 2020). During this process, it turned out that the dimension *Digital teaching and learning* could not be mapped empirically. This is particularly due to the target group of the first survey. Since it was directed at school management members, the focus was placed more on the dimensions of organizational and personnel development, while instructional development was only examined in a rudimentary way.

This resulted in a total of five MMOE dimensions being used to answer the second and third questions: The first dimension, *Equipment and Technology*, was covered by one item dealing with the satisfaction of the same. The *Strategy and Leadership* dimension was operationalized with a three-item scale. In addition to the satisfaction with the school’s digital strategy, the items used related to the structural framework of the school, i.e., the extent to which teachers are offered incentives by school management to adapt their teaching to digital transformation or to what extent they are offered opportunities to acquire digital competencies. The *Organization* dimension was operationalized with two subscales of three items each. The *Pedagogical IT Support* scale comprises content aspects relating to the provision of relief hours for planning instructional innovations with digital teaching and learning methods, the introduction of working groups on pedagogical, digital innovations and the overall satisfaction with pedagogical support in the school. The second subscale *Technological IT Support* includes aspects of satisfaction, availability of first-level support (technological support within ten minutes), and sufficient technical support for the school’s use of teachers’ own devices. The *Employees* dimension was mapped with two subscales and an additional single item. The first scale consists of four items and addresses *teachers’ Digital Competencies*. The items relate to different competence facets, such as application competence, informatics competence, competencies in the area of data protection, and rules of conduct on the topic of IT security. The other two scales can be assigned to the area of attitudes. The *negative Attitudes*, i.e., the fear of doing something wrong when using digital technologies, of reaching one’s own limits or of worrying that digitization may overwhelm one, were operationalized with three corresponding items. The *positive Attitudes* were mapped with one single item on how the respondents feel about digitally supported teaching and learning methods. The last of the five dimensions, *Culture*, was also mapped with one single item, dealing with the perceived acceptance of digital teaching and learning methods among the teaching staff. Table 3 shows the scales with illustrative items as well as the single items used according to their assignment to the dimensions of the MMOE. All scales show acceptable to good Cronbach’s alphas.

**Table 3:** Operationalization and reliability analysis

Variable / Scale (Number of Items)	M (SD)	Alpha	Illustrative Items
*Equipment and Technology*			
*Satisfaction with infrastructure and equipment* (1)^1^	4.53 (1.15)	---	How satisfied are you with the digital infrastructure and equipment in your school?
*Strategy and Leadership*			
*Scale: Strategy and Leadership* (3)^2^	4.10 (1.04)	0.78	How satisfied are you with your school’s digital strategy?
*Organization*			
*Pedagogical IT Support* (3)^2^	3.64 (1.10)	0.68	Our school provides release hours for planning instructional innovations with digital teaching-learning methods.
*Technological IT Support* (3)^3^	3.90 (1.14)	0.82	There is sufficient technical support for the school’s use of teachers’ own devices.
*Employees*			
*Digital competencies of teaching staff* (4)^3^	3.80 (0.85)	0.71	Application competence (e.g., use of Microsoft Office, communication via e-mail, etc.)
*Attitude (Fear)* (3)^4^	2.58 (1.12)	0.87	The idea of doing something wrong when using digital technologies scares me.
*Attitude (Positive)* (1)^4^	4.48 (1.09)	---	I am generally positive about digitally supported teaching and learning methods.
*Culture*			
*Acceptance of digital teaching and learning methods* (1)^5^	4.24 (0.96)	---	How do you assess the acceptance of digital teaching and learning methods among the teaching staff at your school?

*Note.* M: Mean, SD: Standard Deviation, α: Cronbach’s alpha, 6-point Likert scale

^1^ 1 ‘Not at all satisfied’ to 6 ‘Very satisfied’

^2^ 1 ‘Not at all satisfied’ to 6 ‘Very satisfied’; 1 ‘Does not apply at all’ to 6 ‘Fully applies’

^3^ 1 ‘Not at all satisfied’ to 6 ‘Very satisfied’; 1 ‘Do not agree at all’ to 6 ‘Totally agree’

^4^ 1 ‘Do not agree at all’ to 6 ‘Totally agree’

^5^ 1 ‘Very low acceptance’ to 6 ‘Very high acceptance’

### Analysis

To answer the research questions, t-tests for independent samples as well as two linear regression models for both school management members and teachers were used. Since the participants in the study were from different schools (n = 62), independent and identically distributed data could not be assumed with certainty. For this reason, a standard error correction was performed using the statistics software STATA 15. Robust standard errors account for this multilevel structure and filter out potential cluster-related effects (McNeish et al., 2017). Missing values were not imputed. Accordingly, the multivariate analyses were subject to the principle of listwise deletion.

## Results

First, we explored the question of the extent to which perceptions differ between school management members and teaching staff regarding the various dimensions of digital transformation. Due to sample sizes of n > 30 in both subgroups (school management members and teachers), the parametric test procedures could be performed despite a violation of the normal distribution assumption (Field, 2009). A detailed overview of the results is presented in Table 4.

**Table 4:** T-tests between school management members and teachers on various aspects of digital transformation

*Dependent Variable*	*SM* *M (SD)*	*TS* *M (SD)*	*T*	*p*	*d*
*Development State of the Digital Transformation*	4.09 (0.93)	4.32 (1.05)	− 3.355	0.001	− 0.228
*Equipment and Technology*	4.42 (1.03)	4.54 (1.16)	− 1.492	n.s.	---
*Strategy and Leadership*	4.31 (0.94)	4.08 (1.04)	3.103	0.002	0.218
*Organization: Pedagogical IT Support*	3.84 (0.97)	3.62 (1.12)	2.925	0.004	0.199
*Organization: Technological IT Support*	3.92 (1.12)	3.90 (1.14)	0.239	n.s.	---
*Employees: Digital Competencies*	3.58 (0.69)	3.82 (0.86)	− 4.354	0.000	− 0.278
*Employees: Attitudes (fear)*	2.45 (0.94)	2.60 (1.14)	− 2.018	0.045	− 0.133
*Employees: Attitudes (positive)*	3.81 (0.78)	4.55 (1.09)	− 11.635	0.000	− 0.688
*Culture*	4.49 (0.62)	4.65 (0.82)	− 3.262	0.001	− 0.201

*Note.* SM = School Management; TS = Teaching Staff; M = Mean; SD = Standard Deviation; measured with 6-point Likert scales

The analyses identified significant differences between the school management members and the teaching staff in various aspects of the digital transformation. Only the variables *Equipment and Technology* and the subscale *Technological IT Support*, which can be assigned to the content dimension *Organization*, showed no significant effects. The findings also indicate that *Pedagogical IT Support* (d = 0.199) and *Strategy and Leadership* (d = 0.218) were rated better by school management members than by teachers. The reverse was true for the basic assessment of the *Developmental State of Digital Transformation* (d = 0.228), *Digital Competencies of the Teaching Staff* (d = 0.278), *Attitudes (Fear)* (d = 0.133), and *Culture* (d = 0.201). According to Ellis (2010), these effects can be classified as small. By contrast, a large effect can be found for the variable *Attitudes (Positive)*, which was operationalized using the item *“[Teachers are] I am generally positive about digitally supported teaching and learning methods.”* (d = 0.688). The *positive Attitudes* of teachers toward digitally supported teaching and learning methods was rated significantly higher by the teaching staff than by the members of the school management.

The second research question examined whether the model of Ifenthaler and Egloffstein (2020) could be validated empirically, that is, the extent to which the individual content dimensions are related to the general state of development of the digital transformation. Thus, we also assessed whether different effects could be identified between school management members and teachers. A multivariate approach in the form of linear regression analysis was chosen for this purpose. Table 5 presents the regression model for school management members. Table 6 presents the same regression model for the teaching staff.

**Table 5:** Regression Model I – School Management Members (DV: Development State of Digital Transformation)

	*B*	*Robust* *SE*	*Beta*	*T*	*p*
*Equipment and Technology*	*0.247*	*0.068*	*0.281*	*3.60*	*0.001*
*Strategy and Leadership*	0.123	0.082	0.128	1.50	0.140
*Organization: Pedagogical IT Support*	− 0.047	0.078	− 0.049	− 0.60	0.548
*Organization: Technological IT Support*	0.064	0.081	0.081	0.79	0.431
*Employees: Digital Competencies*	*0.239*	*0.111*	*0.180*	*2.16*	*0.035*
*Employees: Attitudes (Fear)*	− 0.039	0.072	− 0.041	− 0.55	0.586
*Employees: Attitudes (positive)*	− 0.036	0.102	− 0.032	− 0.35	0.727
*Culture*	0.274	0.140	0.191	1.96	0.055
Gender^a^	0.020	0.147	0.009	0.13	0.894

R² = 0.339; n = 184; F (9; 60) = 9.88; p < .001; ^a^ 1 ‘male’ 2 ‘female’

**Table 6:** Regression Model II – Teaching Staff (DV: Development State of Digital Transformation)

	*B*	*Robust* *SE*	*Beta*	*T*	*p*
*Equipment and Technology*	*0.288*	*0.031*	*0.319*	*9.31*	*0.000*
*Strategy and Leadership*	*0.485*	*0.033*	*0.483*	*14.58*	*0.000*
*Organization: Pedagogical IT Support*	− 0.046	0.030	− 0.050	− 1.53	0.132
*Organization: Technological IT Support*	− 0.030	0.024	− 0.033	− 1.24	0.219
*Employees: Digital Competencies*	*0.086*	*0.026*	*0.071*	*3.37*	*0.001*
*Employees: Attitudes (Fear)*	*0.061*	*0.021*	*0.066*	*2.97*	*0.004*
*Employees: Attitudes (positive)*	− 0.007	0.018	− 0.007	− 0.38	0.703
*Culture*	*0.126*	*0.034*	*0.099*	*3.72*	*0.000*
Gender^a^	0.035	0.025	0.027	1.40	0.167

R² = 0.509; n = 1.697; F (9; 61) = 132.60; p < .001; ^a^ 1 ‘male’ 2 ‘female’

With an explained variance of R² = 0.339, regression Model I shows a large effect (Ellis, 2010). A closer look at the predictors indicates that both *Equipment and Technology* (β = 0.281) and the subscale *Digital Competencies* (β = 0.180) of the *Employees* dimension were significant contributors to the variance explanation in the dependent variable. The *Culture* factor (β = 0.191) also made a significant contribution at an α = 10% level, indicating a statistical tendency here. The beta coefficients of the significant predictors indicate small to medium effects.

Compared to the first model, which was calculated only with the subsample of school management members, regression Model II (Table 6), in which only the teachers were considered, showed an even higher variance explanation (R² = 0.509). This can also be classified as a large effect or a high model quality. Considering the individual predictors, again *Equipment and Technology* (β = 0.319) and *Digital Competencies* (β = 0.071) were significant contributors to the variance explanation. Although the effect was slightly higher for *Equipment and Technology* compared to Model I, it decreased to a small effect for *Digital Competencies*. The variable *Culture* (β = 0.099), for which a statistical trend was found in Model I, was also significant in Model II, with a small effect.

In contrast to the school management members, however, two key differences emerged in regression Model II (teaching staff). First, the predictor *Attitudes (Fear)* (β = 0.066) showed a significant result, with a small effect. Second, there was a major change in the *Strategy and Leadership* variable (β = 0.483) between the models. Although it did not make a significant contribution to the variance explanation in the first model for the school management members, *Strategy and Leadership* showed up here as a significant predictor, with the largest effect size of all variables included in the model. The effect can be classified as large (Ellis, 2010). Compared to the school management members, it is thus clear that *Strategy and Leadership* had a significant influence on the perceived state of development of digital transformation among the teachers surveyed.

## Discussion

The overarching goal of our study was to assess the digitization-related development state of vocational schools in Switzerland. Through the cooperation of the three institutions involved, it was possible to look at both the perspective of school management members and that of teachers on various aspects of digital transformation.

The first research question of this study sought to define the status quo in the development of transformation in Swiss vocational schools. The assessment of the general development state of the digital transformation revealed that teachers assessed the status quo somewhat better than school management members; therefore, both groups perceived it as advanced. This is in line with the conclusions of the report of Educa (2021), which ascribe a comparatively positive picture to vocational schools regarding the digital transformation, although differences between individual schools undoubtedly exist. The differentiated perception can have various reasons. One possible reason for the digitalization-related status quo could be the different range of tasks performed by school management members compared to teachers. The role of school management members is significantly more complex and overarching, as they must look at the school from a holistic perspective. In addition to classroom activities, this includes, among other things, strategic or personnel aspects that can be assigned to the area of leadership. Furthermore, it is the duty of school management members to consider external influences, such as digitalization-related decisions of the education policy, and to account for and implement necessary measures in the best possible way. In the sense of Fend’s (2008) multi-level theory, school management must keep an eye on developments at the macro, meso, and micro levels. A higher number of task areas increases the likelihood that digitalization-related weaknesses will be noticed more and with a higher granularity. It can be assumed that teachers primarily focus on what happens in the classroom, where strategic or organizational aspects of the digital transformation are not addressed so strongly (primary focus on the micro level).

Another reason for the different perceptions could be that school management members serve as a point of contact for many teachers when digitalization-related aspects are not going as well as they should. The school management is regularly informed of ICT malfunctions by the teachers; however, school management is given very little feedback when ICT works. It is therefore possible that school management has an incorrect perception of the functioning of ICT. Further, it is also up to the school management if some teachers express complete reservation toward the digital transformation or reject it as such. Although this situation certainly has an impact on the school as a whole, it has no direct effect on individual teachers in their day-to-day work. Overall, it can be assumed that teachers are more likely to approach school management members with negative aspects and problems, and that school management is not necessarily as strongly confronted with positive aspects regarding digital transformation.

We must also question whether it might be socially desirable for school management members to be more reticent about the general level of digital development so that development processes can continue. However, a social desirability effect could be present in the teachers’ responses, as they might feel directly exposed. In this regard, the idea of a distributed responsibility for the school management could have led to limit a possible overestimation of the state of the art. Additionally, school management could serve as a kind of “critical friend” who identifies optimization potential in the dimensions of digital transformation and develops these aspects further.

Differences between the two groups can also be identified by considering the individual dimensions of digital transformation. Whereas the areas of *Pedagogical IT Support* and *Strategy and Leadership* were perceived better by the school management members, the opposite was true for the dimensions of *Digital Competencies of the teaching staff*, *Attitudes*, and *Culture*. One possible reason for the differentiated perceptions is the self-assessment or external assessment of these aspects. Individuals with high self-esteem tend to evaluate themselves more positively than others do (e.g., Atwater & Yammarino, 1992; Nilsen & Campbell, 1993; Lindeman et al., 1995). For example, it seems plausible that members of the school management assessed *Pedagogical IT Support* and *Strategy and Leadership* better, as these can be assigned to organizational development, for which the school management members are primarily responsible. Conversely, aspects such as *Digital Competencies* or the *Attitudes* of teachers are better assessed by the same. The different perceptions of the digitalization-related school culture may also be due to the situation described above, in which school management members tend to be informed significantly more often about difficulties or shortcomings regarding the digital transformation. Considering the approach by Petko et al. (2018b), it is noteworthy that aspects assigned to *school readiness* are rated better by school management, while those assigned to *teacher readiness* are rated better by teachers.

The second research question investigated the differences between the school management members and teachers regarding the various dimensions of the MMOE. In this respect, we found that certain dimensions of the MMOE are generally well suited for explaining the general digitalization-related state of development. *Equipment and Technology*, *Digital Competencies*, and *Culture* appeared to be of particular importance. In contrast to the regression model for school management members, however, the greatest effect in the model for teachers was observed for the variable *Strategy and Leadership*. It therefore appears that the *Strategy and Leadership* dimension is a very important aspect from the perspective of the teachers regarding the general status quo of digital transformation. Conversely, this circumstance did not seem to be the case for the school management members themselves. In an attempt to elaborate characteristics of so-called “digital optimal schools”, Eickelmann and Drossel (2020) identify satisfaction with digital infrastructure and equipment, advanced training for teachers, and IT support as the most important factors. While the first two aspects also appear to be important in our study, insofar as we see digitization-related advanced training in the context of increasing digital competencies, with our data we cannot confirm the importance of IT support for the perception of the digitization-related state of development. Regarding the aspects related to the *Strategy and Leadership* dimension, the authors do not make any explicit statements. For the successful design of digitalization-related school development processes, however, it is important that school management members are aware of their special role, in particular regarding their perceived importance in digital transformation processes from the teachers’ perspective. It is their task to drive and sustain digital transformation through appropriate transformational leadership. Without a corresponding commitment on the part of school management members and also considering sufficient organisational resources, the transformation cannot succeed. This is in line with the argumentation of Seufert and Tarantini (2022), who also emphasize the importance of school management members or leadership and highlight their significant role in the development of a culture conducive to innovation as well as the proactive design of change processes. At the same time, it must be considered that school management members also have organizational hurdles to overcome and are constrained by certain restrictions, for example in budgeting or a lack of culture for project-base work in some school environments. This may result in the situation that certain measures can only be implemented to a limited extent, which can diminish the influence of the school management to a certain degree.

We deduced from the results that the inclusion of all school stakeholders – especially school management members and teachers – is indispensable to successfully shape the digital transformation of schools. Scholkmann (2021) also identifies the “individual” (i.e., in our context, both school management members and teachers) as the problem source and at the same time solution regarding sustainable change. Following Graf-Schlattmann et al. (2019, p. 19), they state that a “collective willingness to change” is needed to drive digital transformation. Thus, only a collaborative approach that involves all relevant school stakeholders will result in a successful digital transformation. Stronger communication could be the key to developing a common understanding of digital transformation and, building on this, to further developing the various digital dimensions collaboratively. In this context, Tołwińska (2021) noted that the quality of work in schools can be improved if teachers contribute their knowledge and skills to these processes. The digital competencies of teachers play an important role in related change processes, since teachers who have a higher level of digital competencies perceive the ease of use of ICT (and consequently its usefulness) better (Baturay et al., 2017). It is the task of school management members to promote this exchange by getting teachers to be proactive and not afraid of making mistakes. Thus, collaboration between school stakeholders is perhaps the most important aspect to consider in principle in change processes, and digital transformation as well.

## Limitations

For an adequate interpretation of the results, it is necessary to address the limitations of the study. First, the different sample sizes within the two subgroups, which was to be expected given the significantly smaller population of school management members, need to be mentioned. Nevertheless, the relatively small number of school management members per school means that analyses at the individual school level are only possible to a limited extent. Furthermore, we cannot exclude the possibility of positive selection regarding the sample in both surveys. Participation in the study was voluntary for both school management members and teachers, which means that a corresponding bias seems possible. Moreover, both school management members and teachers from certain schools were more strongly represented in the sample than others. In the multivariate analyses, this clustering problem was accounted for using standard error correction. Another aspect connected to the sample itself, with special regard to vocational education and training, is that companies also represent an important stakeholder in digital transformation processes in vocational schools. Assessing their role and their perspective on digital transformation in vocational schools could be one focal point of future research.

The second key limitation of the study relates to the measurement of selected characteristics regarding self-assessment or social desirability. It can be assumed that when asked about their leadership behavior, school management members can choose response behaviors that are socially expected, which can lead to bias. Further, the survey contained aspects such as the digital competencies of the teachers, which can be better measured by using a corresponding competency test that could afford an increase in both reliability and validity. All measured variables are perceptions of the school management members or teachers; it is therefore not an objective assessment of the digitization-related state of development based on external standards (Educa, 2021).

Another aspect that needs to be mentioned in this context is the difficulty of operationalizing the underlying MMOE (Ifenthaler & Egloffstein, 2020); while the individual dimensions of the model are very complex in terms of content, these cannot be viewed separately from one another. For example, the dimension of *Culture* has many interfaces with other dimensions, such as those of *Employees*. In this context, our operationalization of the model is limited to a certain extent, as the respective theoretical dimensions are not fully mapped. Against the background of our findings, the question arises as to possible limitations regarding the chosen theoretical framework. The results suggest that the dimensions of the model may not have equal value, i.e., individual dimensions may appear more important than others. In addition, there is a possibility that important content aspects are not included in the model. One example could be the establishment of a feedback culture, which might play an important role in successfully shaping the digital transformation (cf. Delcker & Ifenthaler, 2022).

Further, the individual project-related organizational conditions meant that the surveys could not take place during the same period. This circumstance was further compounded by the fact that the survey of school management members was conducted before, and the survey of teachers shortly after the onset of the COVID-19 pandemic. During this period, the issue of digitalization in schools developed a particular dynamic. The measures resulting from the pandemic may have led to a change in certain aspects related to digitalization. This should also be considered when interpreting the results.

Finally, adding the learners’ perspective in future studies could provide an even broader picture of the relevant school stakeholders. In this context, the SELFIE tool of the European Commission (2022) could – especially regarding the assessment of digital competencies – serve as a basis, since it was developed in accordance with the DigCompEdu Framework (Redecker & Punie, 2017) and already combines the perspectives of school management members, teachers and learners.

## Conclusions and Implications

The first objective of this study was to assess the status quo of digital transformation from the perspective of school management members and teachers at vocational schools in Switzerland. The results showed that both status groups generally assessed the digitalization-related state of development as advanced. However, a small effect was observed in that the teachers rated the status quo slightly better than the school management members did. A detailed examination of various aspects of the digital transformation revealed that the individual dimensions of change were perceived differently by school management members and teachers. In the cases of *Equipment and Technology*, which were rated as good by both groups, and *Technological IT Support*, which was also in the satisfactory range, no significant differences in perception could be identified. Although the school management members showed more positive assessments in the areas of *Strategy and Leadership*, *Pedagogical IT Support*, and fear-related *Attitudes* toward digital transformation, the opposite effects were observed regarding the *Digital Competencies*, the *positive Attitudes* of teachers toward digital transformation, and the anchoring of digital transformation in the school culture. In the case of positive attitudes, the effect could be classified as medium to large.

The second objective of this study was to determine which dimensions of the digital transformation can be used as predictors for assessing the digitalization-related status quo of schools. In the model for the school management members, the digital competencies of the teaching staff were identified as a significant predictor, along with equipment and technology, which showed the greatest effect. Both variables remained significant in the teachers’ model as well, with the effect of digital competencies being reduced due to multivariate correlations. Further, both the fear-related attitudes of the teachers, with a comparatively small effect, as well as the anchoring of the digital transformation in the school culture and the dimension of *Strategy and Leadership*, are predictive for the digitalization-related state of development at a significant level. In the case of *Strategy and Leadership*, the effect can be classified as medium to large. In other words, we can conclude that the teachers, in contrast to the school management members, perceived *Strategy and Leadership* as a significant factor regarding the assessment of the digitalization-related development status. This suggests a different level of importance for this dimension in the context of digitalization between the two groups. However, the relevance of this dimension is enormous, as teachers perceive the willingness of school management to promote innovation or change as one of the strongest predictors of school quality (Bonsen et al., 2002; Imboden, 2017).

In summary, our study shows, on the one hand, that the different dimensions of digital transformation are perceived differently by the two observed status groups. On the other hand, some of these dimensions are considered more important than others regarding the perception of the general digitization-related status quo. In this context, differences between school management members and teachers could also be identified. In principle, we focused on obtaining a holistic view of digital transformation by both school management members and, in particular, by teachers, whereas in many other studies the content focus of studies with teachers is often reduced to instructional development, i.e., their digital competencies or the use of ICT in the classroom.

Thus, the question arises as to what implications for the successful design of the digitalization-related school improvement process can be derived from these results. First, it is important that teachers are actively involved in digitalization-related development processes by school management. Based on a jointly developed vision or strategy, common values must be defined that determine both the content-related and the “moral” framework. It is of importance that all school stakeholders agree on what digital transformation means for their school and what part the individual status groups must contribute so that the transformation process can be shaped successfully. A constructive trial-and-error culture can help to quickly solve potential problems and optimize the transformation process (Tołwińska, 2021).

To support these findings, further studies are needed on the digital transformation of vocational schools that look beyond the classroom level to the entire school development process. It is necessary to observe how schools evolve in the different theoretical dimensions of digital transformation, or whether some of these dimensions emerge as more important than others when assessing the digital status quo. In this regard, it would be helpful to consider the individual dimensions in more detail to better examine the interrelationships between them. Additionally, the school management’s leadership competence should also be included in these thoughts, as school management members with advanced leadership competence will find it easier to drive change (Hallinger & Heck, 2010; Bonsen, 2016b). Comparisons between vocational and general education schools, and between different countries can also help identify potential and formulate recommendations for action. It will be interesting to see how digital transformation in schools progresses over time, particularly in light of the complex dynamics of the topic, which was accelerated by the COVID-19 pandemic.
